# CD63/81 Small Extracellular Vesicles in the Aqueous Humor are Retinoblastoma Associated

**DOI:** 10.1167/iovs.64.10.5

**Published:** 2023-07-06

**Authors:** Sarah Pike, Chen-Ching Peng, Paolo Neviani, Jesse L. Berry, Liya Xu

**Affiliations:** 1 The Vision Center, Children's Hospital Los Angeles, Los Angeles, California, United States; 2 USC Roski Eye Institute, Keck School of Medicine of the University of Southern California, Los Angeles, California, United States; 3 Extracellular Vesicle Core, Children's Hospital Los Angeles, Los Angeles, California, United States; 4 The Saban Research Institute, Children's Hospital Los Angeles, Los Angeles, California, United States; 5 Norris Comprehensive Cancer Center, Keck School of Medicine of the University of Southern California, Los Angeles, California, United States

**Keywords:** extracellular vesicles (EVs), retinoblastoma, aqueous humor (AH), liquid biopsy

## Abstract

**Purpose:**

Although biopsy is contraindicated in retinoblastoma (RB), the aqueous humor (AH) is a robust liquid biopsy source of molecular tumor information, facilitating biomarker discovery. Small extracellular vesicles (sEVs), promising biomarker candidates across multiple cancers, were recently identified in RB AH, but relationships between sEVs and RB clinical features are unknown.

**Methods:**

We analyzed sEVs in 37 AH samples from 18 RB eyes of varying International Intraocular Retinoblastoma Classification (IIRC) groups and explored clinical correlations. Ten samples were collected at diagnosis (DX) and 27 during treatment (Tx). Unprocessed AH underwent Single Particle-Interferometric Reflectance Imaging Sensor (SP-IRIS) analysis for fluorescent particle count and tetraspanin immunophenotyping; counts were subsequentially converted to percentages for analysis.

**Results:**

Comparing DX and Tx samples, a higher percentage of CD63/81+ sEVs was found in DX AH (16.3 ± 11.6% vs. 5.49 ± 3.67% *P* = 0.0009), with a more homogenous mono-CD63+ sEV population seen in Tx AH (43.5 ± 14.7% vs. 28.8 ± 9.38%, *P* = 0.0073). Among DX samples, CD63/81+ sEVs were most abundant in group E eyes (*n* = 2) compared to group D (*n* = 6) by count (2.75 × 10^5^ ± 3.40 × 10^5^ vs. 5.95 × 10^3^ ± 8.16 × 10^3^, *P* = 0.0006), and to group A + B (*n* = 2) by count (2.75 × 10^5^ ± 3.40 × 10^5^ vs. 2.73 × 10^2^ ± 2.59 × 10^2^, *P* = 0.0096) and percentage (32.1 ± 7.98% vs. 7.79 ± 0.02%, *P* = 0.0187).

**Conclusions:**

CD63/81+ sEVs enrich AH from RB eyes before treatment and those with more significant tumor burden, suggesting they are tumor-derived. Future research into their cargo may reveal mechanisms of cellular communication via sEVs in RB and novel biomarkers.

Although retinoblastoma (RB) is the most common pediatric intraocular malignancy,^1^ the ability to diagnose and prognosticate this cancer is limited by a strict contraindication to tumor biopsy.^2^^–^^8^ Because of this, subjective examination and imaging is used for diagnosis, grouping of tumors by International Intraocular Retinoblastoma Classification (IIRC) guidelines, and designing treatment plans. Aqueous humor (AH), the clear fluid in the anterior chamber, has been established as a liquid biopsy source of molecular tumor information^9^^–^^13^ that can be safely collected at diagnosis and during treatment for RB.^11^^,^^14^^,^^15^ Through analysis of cell-free DNA (cfDNA) in AH from RB eyes, in vivo diagnostic and prognostic biomarkers have been established.^9^^,^^10^^,^^12^^,^^16^^,^^17^ However, use of cfDNA-based biomarkers has limitations. These include reliance on the presence of somatic copy number alterations (SCNAs) in tumor DNA, which are not universally found,^12^ and the significant decrease in AH cfDNA concentration that occurs with treatment,^18^ which limits longitudinal utility.^19^ Therefore, the search for abundant, stable, and informative RB analytes in the AH continues.

One such analyte, of particular interest in cancer research, is extracellular vesicles (EVs). EVs are membrane-enclosed vesicles secreted by all cell types^20^ detectable in all body fluids, including the ocular fluids AH, vitreous humor, and tears.^21^ EVs are crucial participants in cellular communication, immune modulation, and physiologic homeostasis through the removal of excess or unnecessary cellular material.^21^^,^^22^ They deliver signals to target recipient cells through the transfer of their cargo, with the potential to cause cellular reprogramming of the recipient cells.^23^ EV cargo includes bioactive molecules, such as nucleic acids, lipids, metabolites, and cytosolic proteins which correlate with the molecular information of their parent cells.^21^^–^^24^ The lipid bilayer membrane of EVs contributes to their physiologic stability making them ideal biomarker candidates.^22^^,^^25^

EVs are broadly classified by size^26^ with small EVs (sEVs) ranging between 30 and 150 nm and large EVs from 100 nm to 1 µm.^27^ Large EVs are ectosomes derived from plasma membrane budding, whereas the vast majority of sEVs are of endosomal origin (also known as exosomes) with fewer ectosomes of this size.^22^ EVs have surface proteins, including tetraspanins (CD63, CD81, and CD9), which have been established as the most commonly found exosomal sEV surface biomarkers that can be used for phenotypic expression profiling.^22^^,^^24^^,^^26^^,^^28^ Tetraspanins play a crucial role in the biogenesis and function of EVs by regulating their formation, cargo sorting, and interaction with target cells.^29^^,^^30^ They are involved in EV subtype specificity, enabling the formation of distinct subpopulations with unique protein composition and functional properties.^29^ However, their contribution to EV subtypes is still not fully understood, and further research is needed to uncover the precise mechanisms and limitations of tetraspanins in shaping EV functionality.

Small EVs are the most well described in the setting of cancer^27^ and are also the predominant size found in intraocular biofluids.^31^^–^^35^ For cancer, tumor-derived sEVs have been shown to promote tumor formation, progression, treatment resistance, and metastases^24^^,^^36^^,^^37^ allowing for the discovery of diagnostic and prognostic biomarkers for multiple cancer types, including breast,^38^ lung,^39^^,^^40^ gastric,^36^ and melanoma,^41^ among others.^24^ In the eyes, sEVs have been investigated in diseases including age-related macular degeneration, retinal vascular disease, glaucoma, uveitis, and thyroid eye disease, primarily through analysis of tissue, cell lines, and serum, whereas AH studies are limited.^21^^,^^25^^,^^35^ For RB, research is being done to isolate sEVs from tumor cell lines and patient serum for vesicle characterization and cargo analysis^42^^–^^44^ which has yielded meaningful data, supporting sEVs as a possible biomarker for RB. Although sEVs have been explored in ocular diseases, their potential as a biomarker for disease and their dynamics in the AH are areas of limited research,^33^^,^^45^^–^^49^ with none examining RB specifically.

Our laboratory recently demonstrated that tetraspanin positive sEVs are detectable in a small cohort of pediatric AH collected from eyes with congenital cataract, congenital glaucoma, pediatric retinal disease, and RB.^50^ Analysis of AH was done with Single Particle-Interferometric Reflectance Imaging Sensor (SP-IRIS) technology, which examines the sEV subpopulations at a single vesicle resolution. We found distinct tetraspanin expression patterns with specific enrichment of mono-CD63+ sEVs in the AH, suggesting this may be the dominant AH sEV subtype. The AH specificity of mono-CD63+ sEVs is supported in recent literature, with AH from pseudoexfoliation eyes also exhibiting mono-CD63+ dominance.^47^ Although we identified sEVs in AH from RB eyes, and observed increased tetraspanin heterogeneity in diagnostic RB AH samples compared to AH samples from treated RB eyes,^50^ the relationships between sEV expression profiles and RB clinical features remain unknown. Characterizing these relationships is important in evaluating the potential of sEVs as a biomarker for RB. Herein, we explored sEV dynamics in AH samples from patients with RB with various IIRC disease classifications, collected at multiple treatment time points, to look for clinical correlations.

## Methods

### Patients

This study was conducted under the Institutional Review Board approval at Children's Hospital Los Angeles (CHLA), and it conformed to the requirements of the United States Insurance and Privacy Act and to the tenants of the Declaration of Helsinki. All patients included in the analysis provided written informed consent for a biorepository at CHLA via a legal guardian. AH data were kept separate from clinical data until final retrospective analysis.

### Sample Collection

As previously described in detail,^11^^,^^50^ clear corneal paracentesis was performed to extract up to 0.1 mL of AH as part of a routine procedure for anterior segment surgery at diagnosis or during treatment. Samples were taken from enucleated eyes immediately after enucleation. Treatment was done per CHLA protocol^51^^–^^53^ in a nonrandomized manner, and treating physicians had no knowledge of the AH analysis results. Samples were aliquoted and stored at −80°C until analysis.^11^

### Single Particle-Interferometric Reflectance Imaging Sensor Analysis

Single Particle-Interferometric Reflectance Imaging Sensor (SP-IRIS) technology allows for phenotyping and digital quantification of various populations of individual EVs in sample by using fluorescently tagged immunocapture antibodies targeted against surface marker tetraspanin proteins. In identifying EVs using tetraspanin protein expression, we attempted to profile typical exosomal sEVs without targeting typical similarly sized ectosomes. Here, SP-IRIS by ExoView R100 analysis was performed with the ExoView Human Tetraspanin Kit (Unchained Labs, Pleasanton, CA, USA) as published.^50^ In brief, a desired volume of sample was obtained using 0.25 to 10 µL of unprocessed AH diluted in buffer A to a final volume of 40 µL. Then, 35 µL of the sample was incubated on a ExoView Tetraspanin Chip for 16 hours. Chips were then washed and incubated with immunocapture antibodies (anti-CD9 CF488, anti-CD81 CF555, and anti-CD63 CF647) based on the manufacturer protocol (Unchained Labs). Chips were imaged with the ExoView R100 reader using ExoView Scanner version 3.2 acquisition software; data were analyzed with the ExoView Analyzer version 3.2.

**Figure 1.**
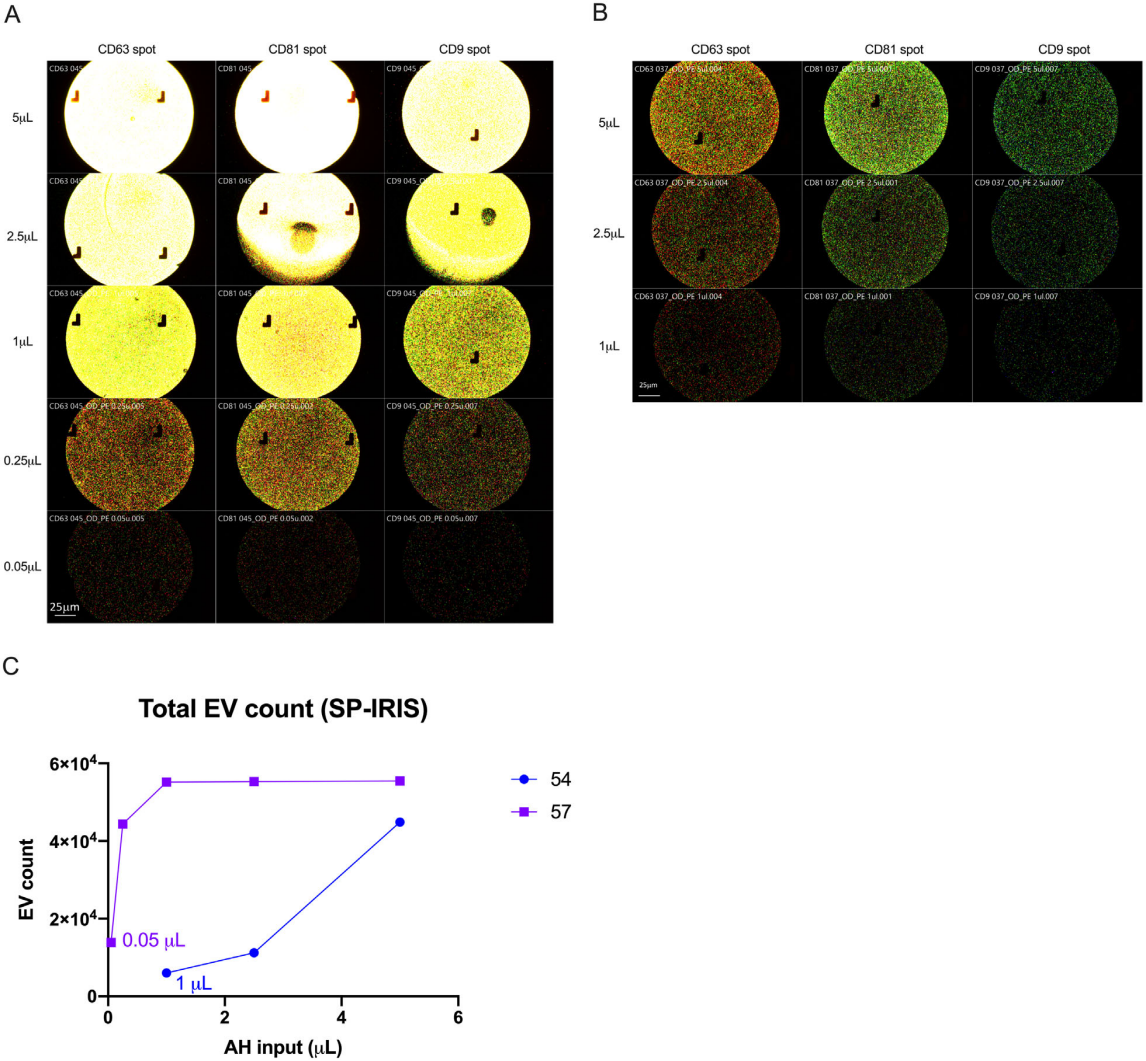
Analytical optimization for SP-IRIS analysis of aqueous humor (AH) by the ExoviewR100 system for tetraspanin expression profiling using 2 unprocessed AH samples at varying input volumes. Representative fluorescent images detected by fluorescent-conjugated antibodies (*red =* CD63-AF647, *green* = CD81-AF555, and *blue* = CD9-AF488). (**A**) ChipView of AH from case 57 with total sample saturation. (**B**) ChipView of AH from case 54 with titrated sample saturation. (**C**) Bar plot displaying EV enumeration (Y axis) based on AH input volume (X axis) for both samples, showing that EV enumeration is linear relative to input volume when the chip is unsaturated, as in case 54 (*blue line*).

Samples were loaded in volumes that ensured particle counts fell within the instrument's linear detection range (200 to 6000 particles per fluorescent channel). To compare the data accurately, the particle counts were normalized to a standardized volume of 10 µL using a dilution factor during the final analysis. For case 57, the input volume was limited to 0.25 µL to keep the captured EV count below 6000 on each chip, avoiding saturation bias (Figs. 1A, 1C, Supplementary Fig. S1A). In the titration of case 54's AH sample, it was observed that EV enumeration remained linear with respect to input volume when the chip was not saturated (Figs. 1B, 1C, Supplementary Fig. S1B). This enabled the comparison of EV counts downstream with adjusted AH volumes, as co-expression patterns were well-preserved among unsaturated inputs, and counts could be normalized using the dilution factor.

### Statistical Analysis

Categorical variables were compared with Fisher's exact test. Continuous variables were summarized as the mean ± standard error of mean (SEM) and average percentages ± SEM Shapiro-Wilk testing was used for distribution of all continuous variables. All data showed non-normal distribution. Non-parametric Mann–Whitney *U* testing was used for all non-normally distributed variables (EV counts and percentages). Analysis of variance testing (ANOVA) testing and Tukey's multiple comparisons were used for EV counts and percentages across IIRC groups. Tetraspanin co-expression percentages were calculated based on the total number of fluorescent particles in the sample per SP-IRIS analysis. All statistical tests were 2-tailed, and *P* < 0.05 was considered statistically significant. Analyses and plots were conducted using Prism 8 (GraphPad).

## Results

### Participant Demographics and Treatment Conditions

**Figure 2.**
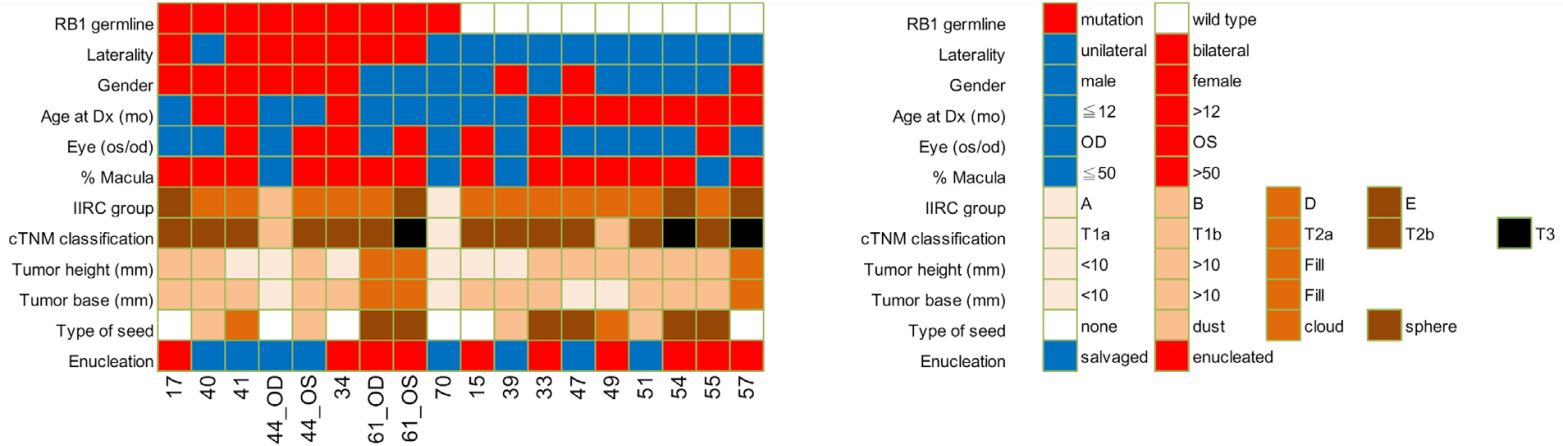
Clinical demographic information for each retinoblastoma eye included in analysis.

A total of 37 RB AH samples from 18 eyes in 16 patients were included in analysis (see the Table). Ten of the AH samples were collected at diagnosis (DX) and the remaining 27 were collected during RB treatment (Tx). Eight male patients and eight female patients were included in the analysis, 10 with unilateral disease and 6 with bilateral disease. Median age at diagnosis was 17 months (range = 2–35 months). By IIRC grouping, one group A, one group B, 12 group D, and 4 group E eyes were included in the analysis. No patients withdrew or were lost to follow-up over the study period. Demographic data for patients with RB can be found in the Table and Figure 2.

**Table.** Clinical Demographic Information for Each Retinoblastoma Eye Included in Analysis

Case ID	Age at Diagnosis (mo)	Sex	IIRC Group	Eye Included in Analysis	Laterality	Germline *RB1* Mutation	Vitreous Seeding Class at Diagnosis	Subretinal Seeding at Diagnosis?	Tumor Growth Pattern	Number of Samples	Time of Sample Collection	Initial Treatment	Outcome
17	2	F	E	OD	B	Positive	None	Yes	Exophytic	1	Tx	IVC	Enucleated
15	10	M	D	OS	U	Negative	None	Yes	Exophytic	1	Tx	IAC	Enucleated
39	4	F	D	OD	U	Negative	Dust	No	Endophytic	3	Tx	IVC	Salvaged
40	18	F	D	OD	U	Positive	Dust	No	Endophytic	5	Tx	IVC	Salvaged
41	19	F	D	OS	B	Positive	Cloud	No	Endophytic	1	Tx	IVC	Salvaged
44^*^	4	F	B	OD	B	Positive	None	Yes	Endophytic	1	DX	IVC, IAC	Salvaged
44^*^	4	F	D	OS	B	Positive	Dust	Yes	Exophytic	1	DX	IVC, IAC	Salvaged
33^*^	22	M	D	OS	U	Negative	Sphere	Yes	Mixed	3	1 DX, 2 Tx	IAC	Enucleated
34^*^	16	F	D	OS	B	Positive	None	Yes	Mixed	1	DX	Enucleation	Enucleated
47^*^	15	F	D	OD	U	Negative	Sphere, some dust	Yes	Mixed	4	1 DX, 3 Tx	IVC	Salvaged
49^*^	35	M	D	OD	U	Negative	Predominately cloud	Yes	Endophytic	1	DX	Enucleation	Enucleated
51	30	M	D	OD	U	Negative	Dust	Yes	Exophytic	1	Tx	IAC	Salvaged
54^*^	25	M	E	OD	U	Negative	Sphere	No	Endophytic	1	DX	Enucleation	Enucleated
61	9	M	D	OD	B	Positive	Sphere	Yes	Endophytic	4	Tx	IVC	Enucleated
61	9	M	E	OS	B	Positive	Sphere	Yes	Endophytic	4	Tx	IVC	Enucleated
55^*^	24	M	D	OS	U	Negative	Sphere	No	Endophytic	3	1 DX, 2 Tx	IAC	Enucleated
57^*^	24	F	E	OD	U	Negative	Unknown	Unknown	Mixed	1	DX	Enucleation	Enucleated
70^*^	2	M	A	OD	B	Positive	None	No	Endophytic	1	DX	Laser	Salvaged

Abbreviations: IIRC, International Intraocular Retinoblastoma Classification; Tx, sample taken during treatment; DX, diagnostic sample taken prior to treatment; IVC, intra-venous (systemic) chemotherapy; IAC, intra-arterial chemotherapy.

* The patient was included in the diagnostic (DX) cohort.

### Tetraspanin Expression Profiling and Quantification Characterized by SP-IRIS Analysis

#### Comparison of sEV Profiles Between Diagnostic and Treatment Active AH Samples

**Figure 3.**
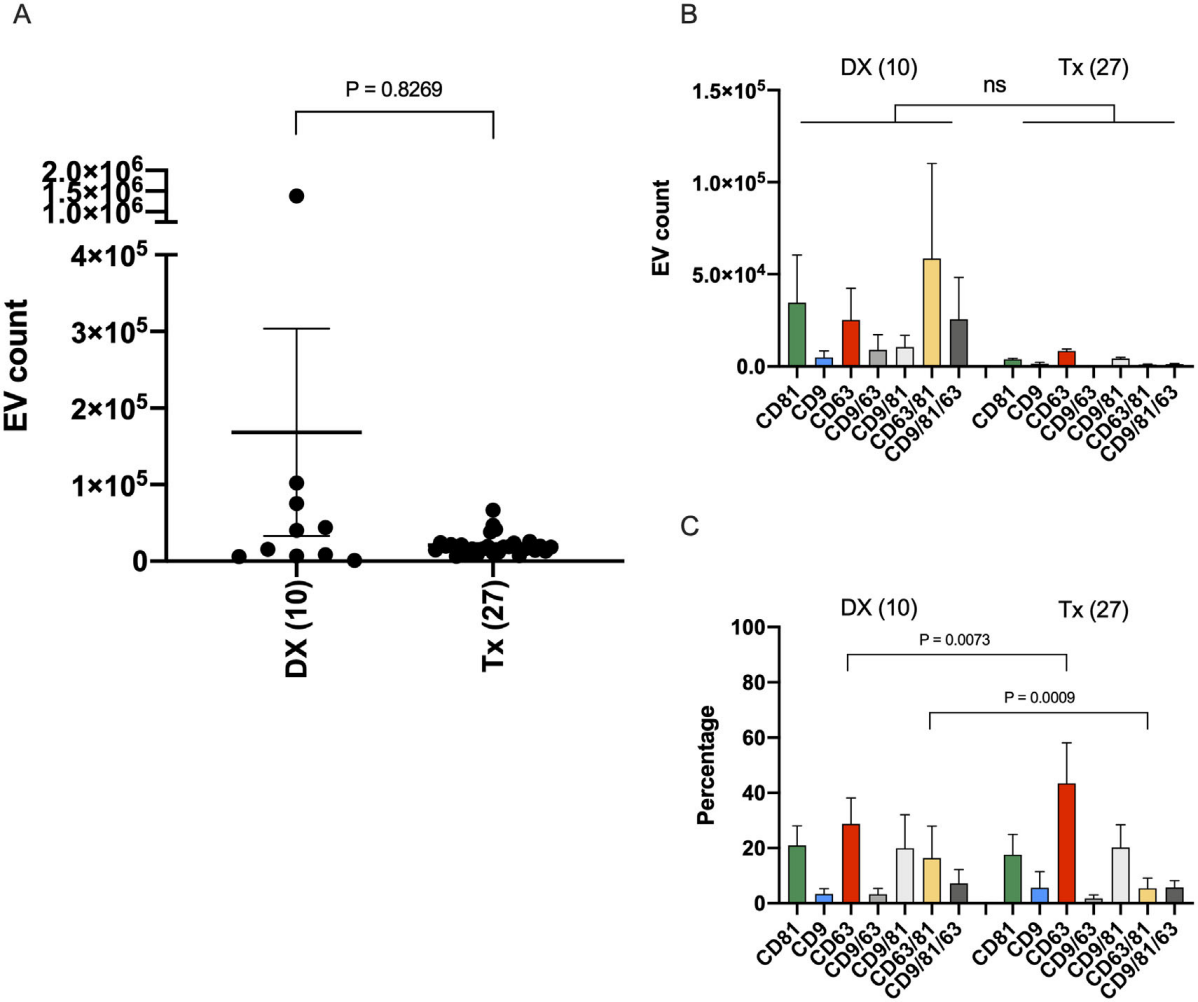
Quantitative comparison of small extracellular vesicle (sEV) tetraspanin subpopulation expression profiles in diagnostic (DX) and treatment active (Tx) unprocessed retinoblastoma (RB) aqueous humor (AH) samples analyzed with SP-IRIS by ExoView R100. (**A**) Mean total sEV counts compared between AH samples taken from DX and Tx eyes. (**B**) Mean sEV subpopulation counts compared between DX and Tx AH. (**C**) Mean sEV subpopulation percentages compared between DX and Tx AH. The *yellow bar* represents the CD63/81+ sEV subpopulation, which is the dominant sEV subtype in AH from RB eyes at diagnosis, and the *red bar* represents the mono-CD63+ sEV subpopulation, which is the dominant sEV subtype in AH from RB eyes collected during treatment. Comparisons made with nonparametric Mann–Whitney *U* testing. Error bars represent ± one SEM.

Quantification of our 37 RB AH samples revealed an average of 1.68 × 10^5^ (±4.28 × 10^5^) tetraspanin-positive sEVs in the DX samples (*n* = 10) and a comparatively lower average of 2.10 × 10^4^ (±1.33 × 10^4^) sEVs in Tx samples (*n* = 27; see Fig. 3A). When we compared immunocaptured sEV subpopulations by count among the groups, a higher average number of all subpopulations was observed in DX samples compared to Tx samples (see Fig. 3B), although differences were not statistically significant due to substantial variations among DX samples. When we compared sEV subpopulations between DX and Tx samples by percentage, we found that DX AH exhibited a heterogenous sEV subpopulation distribution, with a significantly higher subpopulation percentage of CD63/81+ sEVs (DX AH, 16.3 ± 11.6% vs. Tx AH, 5.49 ± 3.67%, *P* = 0.0009; see Fig. 3C). In AH from treated RB eyes, the sEV subpopulation distribution appeared more homogenous, with a significantly higher subpopulation of mono-CD63+ sEVs (Tx AH, 43.5 ± 14.7% vs. DX AH, 28.8 ± 9.38%, *P* = 0.0073; see Fig. 3C).

#### Comparison of sEV Population Profiles Among Diagnostic AH Samples by RB Clinical Features

**Figure 4.**
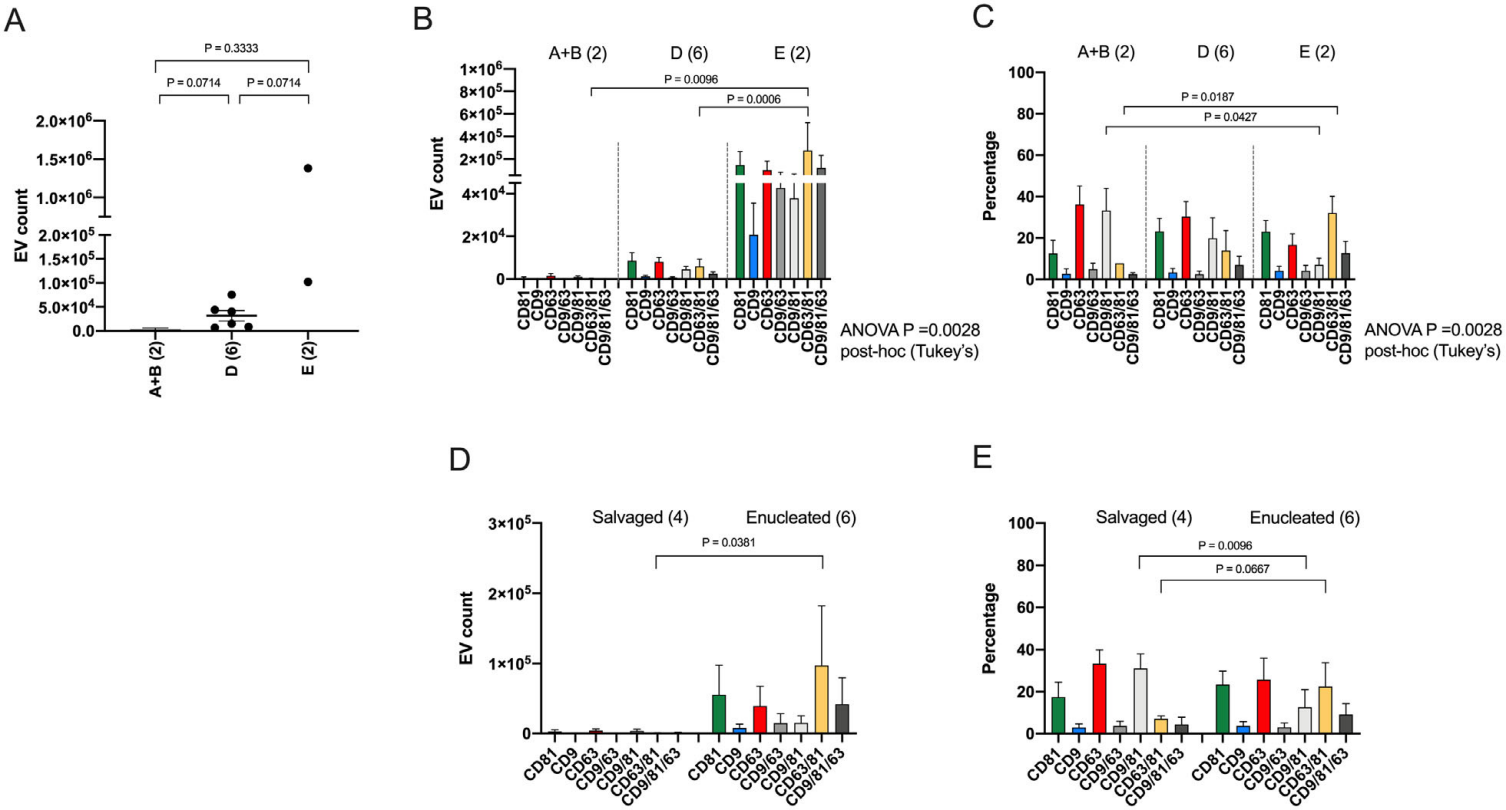
Small extracellular vesicle (sEV) tetraspanin expression profiles in unprocessed diagnostic (DX) retinoblastoma (RB) aqueous humor (AH) samples analyzed using SP-IRIS by ExoView R100. (**A**) Comparison of mean sEV total counts in DX AH samples stratified by International Intraocular Retinoblastoma Classification (IIRC) group of the eye using non-parametric Mann–Whitney *U* analysis. (**B**) Comparison of mean sEV subpopulation counts in DX AH samples stratified by IIRC using ANOVA and Tukey's tests for multiple comparisons. (**C**) Comparison of mean sEV subpopulation percentages between DX AH samples stratified by IIRC using ANOVA and Tukey's tests for multiple comparisons. Note that the CD63/81+ sEV subpopulation percentage (*yellow bar*) increases in a stepwise manner from lower to higher disease burden (**A + B** to **D** to **E**). (**D**) Comparison of mean sEV subpopulation counts in DX AH samples stratified by eye-survival outcome, enucleated versus salvaged, using nonparametric Mann–Whitney *U* analysis. (**E**) Comparison of mean EV subpopulation percentages in DX AH samples stratified by eye-survival outcome, enucleated versus salvaged, using nonparametric Mann–Whitney *U* analysis. As denoted by the *yellow bar*, the CD63/81+ subpopulation is larger in AH collected from eyes which required enucleation due to tumor burden. Error bars represent ± one SEM.

We stratified the 10 DX AH samples by IIRC grouping of the eyes of the sample collection in order to understand differences in sEV populations among the IIRC groups. We combined samples from IIRC groups A and B in analyses given the small tumor sizes in these eyes.^54^ By SP-IRIS analysis, the average total number of immunopositive sEV particles in DX AH samples by IIRC group were 3.51 × 10^3^ (±3.33 × 10^3^) in group A + B samples (*n* = 2), 3.17 × 10^4^ (±2.67 × 10^4^) in group D samples (*n* = 6), and 7.42 × 10^5^ (±9.05 × 10^5^) in group E samples (*n* = 2). When total sEV counts were compared among the IIRC groups, no significant differences were found (see Fig. 4A). However, there were significant differences when comparing sEV subpopulations among groups. When we compared average sEV subpopulation counts among in IIRC groups (see Fig. 4B), we found that CD63/81+ sEVs were significantly more abundant in group E AH (2.75 × 10^5^ ±3.50 × 10^5^) than in group D AH (5.95 × 10^3^ ± 8.16 × 10^3^, *P* = 0.0006) or group A + B AH (2.73 × 10^2^ ± 2.59 × 10^2^; *P* = 0.0096). When we compared sEV subpopulations among groups (see Fig. 4C), CD63/81+ sEVs were higher in most advanced group E AH (32.1 ± 7.98%) compared to least advanced group A + B AH (7.79 ± 0.02%, *P* = 0.0187). CD9/81+ sEV percentages were higher in group A + B AH (33.3 ± 10.7%) than in group E AH (7.06 ± 3.24%, *P* = 0.0427). Next, we compared sEV subpopulations between DX samples based on eye-survival outcomes, enucleated (*n* = 6) versus salvaged (*n* = 4; see the Table, Figs. 4D, 4E). The enucleated group included four eyes enucleated as primary treatment and two eyes secondarily enucleated after treatment failure (see the Table). By sEV count comparison, we found a significantly higher number of CD63/81+ sEVs in AH from enucleated eyes (9.71 × 10^4^ ± 2.09 × 10^5^) compared to AH from salvaged eyes (7.69 × 10^2^ ± 8.49 × 10^2^, *P* = 0.0381; see Fig. 4D). By percentage comparison, we identified a larger percentage of CD63/81+ sEVs in enucleated AH with marginal significance (22.5 ± 11.3%, enucleated versus 7.10 ± 1.41%, salvaged, *P* = 0.0667; see Fig. 4E). Additionally, we saw more CD9/81+ sEVs in salvaged eyes (31.1 ± 6.87%) compared to enucleated eyes (12.6 ± 8.34%, *P* = 0.0096; see Fig. 4E).

## Discussion

We previously published that sEVs can be detected in pediatric AH. This analysis included several ocular disease types and demonstrated a predominantly mono-CD63+ sEV subpopulation in the AH.^50^ Interestingly, the mono-CD63+ sEV subtype is rare in other types of biofluid and is likely specific to the AH. In the current analysis, we evaluated relationships between sEV phenotypic expression profiles and clinical features of RB eyes in order to determine the presence of potential tumor-derived sEVs. We included a cohort of 37 RB AH samples from 18 eyes, 10 of which were collected from eyes at DX prior to therapy. We observed a significantly dominant subpopulation of CD63/81+ sEVs in RB eyes, and we hypothesize that these sEVs are tumor-derived. The tumoral nature of these sEVs is supported by the following evidence:

1) AH samples taken at DX had significantly higher CD63/81+ sEV subpopulations than AH taken during therapy when tumors are smaller and less active, whereas the AH from treated RB eyes was mono-CD63+ sEV dominant.
2) There were significantly more CD63/81+ sEVs in eyes with more advanced disease by IIRC grouping, which are associated with increased tumor burden, as well as in those eyes with abundant or treatment resistant tumors that required enucleation.

Using SP-IRIS analysis, we found that the average total number of immunofluorescent sEVs in AH samples from eyes at DX prior to treatment was eight times higher than the number of sEVs in AH from RB eyes undergoing Tx (see Fig. 3A). When we compared sEV subpopulations between DX and Tx AH samples, we discovered a significantly higher mono-CD63+ sEV subpopulation percentage in Tx AH (see Fig. 3C). This is consistent with our previous sEV study, and indicative of a positive treatment response with the eye approaching a more “normal” ocular state.^50^ In the present study, with more RB AH samples, we were able to identify a dominant subpopulation percentage of CD63/81+ sEVs in DX AH (see Fig. 3C).

The main goal of this study was to evaluate sEV subpopulations in AH from RB eyes to determine the presence of tumor-derived vesicles. When we compared average total sEV counts in DX samples by IIRC group, we found that group E samples had the largest total number of sEVs, and noticed a downtrend in sEV number with decreasing IIRC disease severity (see Fig. 4A). The average total number of sEVs decreased 10 fold among group E eyes, which have the most advanced disease,^54^ and group D eyes; with again a 10 fold decrease in total sEV number between group D eyes and group A + B eyes, which have the least advanced disease.^54^ We compared sEV subpopulation counts and percentages between DX samples by IIRC, and found significantly more CD63/81+ sEVs in group E eyes (see Figs. 4B, 4C). We also compared sEV subpopulations between DX samples based on ultimate eye-survival outcomes, and found more CD63/81+ sEVs in eyes which required enucleation compared to eyes which were salvaged with Tx (see Figs. 4D, 4E). Because the CD63/81+ sEV subpopulation was highest in AH from RB eyes before Tx and, more specifically, in those RB eyes at diagnosis with advanced group E disease and those which required surgical removal due to tumor burden or treatment resistance, we propose that sEVs with this signature are RB tumor-associated.

Small EVs co-expressing CD63 and 81 are reportedly rare in the plasma and serum.^28^^,^^55^^,^^56^ This finding, and the existence of the blood-ocular barrier which ensures immuno-protection of the ocular space, suggests that the CD63/81+ sEV AH subpopulation originates in the eye. We observed a return to mono-CD63+ sEV dominance in RB AH samples after treatment. Mono-CD63+ sEVs are likely AH specific based on our previous study and a study of AH from pseudoexfoliation eyes,^47^^,^^50^ providing further evidence that the CD63/81+ sEV subpopulation may come from diseased ocular tissue. A recent evaluation of sEVs in tears from keratoconus eyes revealed that tear sEVs are neither mono-CD63+ predominant nor CD63/81+ predominant,^57^ reinforcing the intraocular nature of both subpopulations. Small EV membrane protein markers and cargo have been reported to correlate with disease for multiple cancer types, including head and neck, pancreatic, ovarian, and gastric cancers,^58^^–^^61^ so we submit that the existence of an RB-derived sEV population designated by the CD63/81+ tetraspanin signature is possible.

Because our data suggest that the CD63/81+ sEV subpopulation in RB AH may be tumor-derived, we propose that the contents of these sEVs warrant further research. Ragusa et al.^62^ analyzed tetraspanin-positive sEVs in the vitreous humor (VH) of patients with uveal melanoma (UM), an adult intraocular cancer, and found a significant difference in sEV miRNA content compared to controls, suggesting that the transformed melanocytes secrete “oncogenic exosomes” with miRNA cargo that may be influential in affecting the tumor microenvironment.^62^ In investigating the oncogenic potential of EVs from UM cell lines, Tsering et al.^37^ demonstrated the involvement of UM EV protein cargo in cancer progression and metastasis, further emphasizing the importance of EV cargo in tumorigenesis. For RB, investigation of the molecular events which drive tumorigenesis through analysis of circRNA and miRNA in tumor tissue and cell lines^63^ is an active area of research, with exciting discoveries being made. We propose that analysis of CD63/81+ “oncogenic exosome” cargo could contribute to this growing body of molecular RB research and allow for continued prognostic biomarker discovery using an AH liquid biopsy approach. Although in our study the AH sample cohort was robust, our relatively small number of diagnostic AH samples serves as a limitation. We are actively collecting DX AH and plan to continue our CD63/81+ sEV analyses by IIRC group and eye-survival outcome using a larger sample size. Currently, the correlation between sEV phenotype and cargo content necessitates additional investigation in order to gain a deeper understanding. However, our study serves as an important initial step in characterizing RB associated sEVs and lays the groundwork for future research exploring the relationship between sEV phenotype and cargo composition. We are confident that further evaluation of this intriguing association will provide valuable insights and contribute significantly to the field.

In conclusion, we present the phenotypic subpopulation profiles of sEVs in AH samples collected from pediatric RB eyes with varying IIRC classifications and eye-survival outcomes. To this author's knowledge, this is the largest study of sEVs in RB AH to date. We confirmed that sEVs were present in all AH samples by identifying bona fide tetraspanin membrane proteins. Using SP-IRIS analysis, we found that the total number of sEVs in RB AH decreased with treatment. We performed tetraspanin subpopulation profiling of all AH samples, and found a heterogenous sEV distribution in DX AH with significant enrichment of CD63/81+ sEVs, which homogenized with treatment to a predominantly mono-CD63+ population. CD63/81+ sEVs significantly populated diagnostic AH from most advanced group E eyes and those eyes which required surgical removal due to massive tumor involvement or treatment resistance. Because the CD63/81+ sEV subpopulation was increased in RB eyes prior to treatment, we believe these sEVs are tumor-derived. The CD63/81+ sEV subpopulation was also highest in AH from RB eyes with more significant tumor burden, further supporting this hypothesis. From our results, we propose that future research into the cargo of CD63/81+ sEVs could be a vital step in the discovery of RB biomarkers with important clinical implications.

## Supplementary Material

Supplement 1
