# Supplementary material for: CD63/81 Small Extracellular Vesicles in the Aqueous Humor are Retinoblastoma Associated
Source: Invest Ophthalmol Vis Sci. 2023 Jul 6;64(10):5. doi: 10.1167/iovs.64.10.5 (PMC10337798; doi:10.1167/iovs.64.10.5)
Supplement: Supplement 1 [file iovs-64-10-5_s001.pdf]

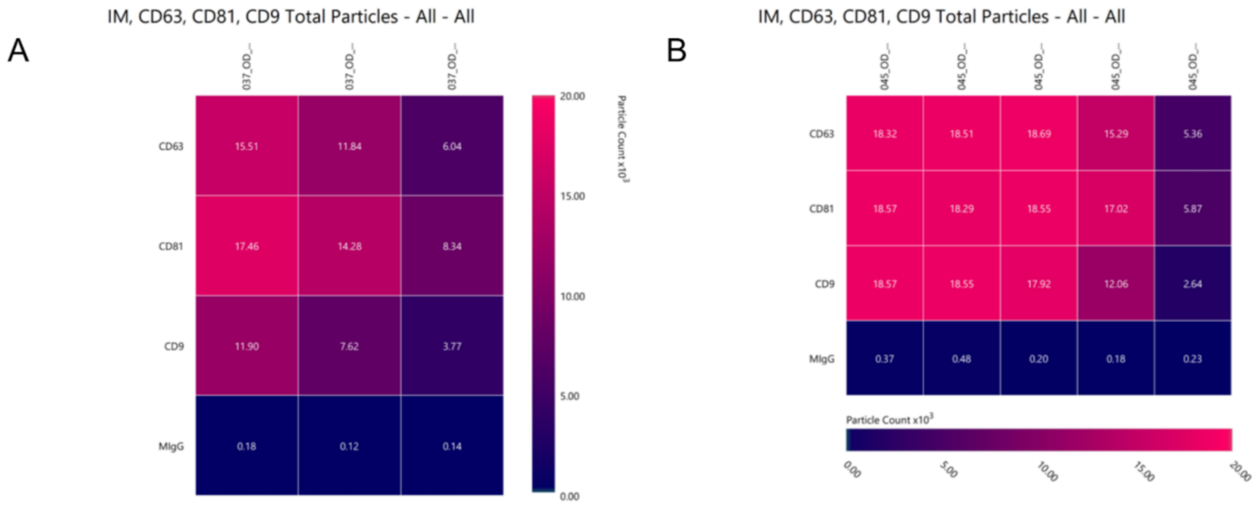

Supplementary Figure 1: Heat maps of tetraspanin expression profiles for two unprocessed aqueous humor samples at varying input volumes generated using SP-IRIS analysis with the ExoviewR100 system. Immunofluorescent images detected by fluorescent-conjugated antibodies (red: CD63-AF647, green: CD81-AF555, and blue: CD9-AF488). (A) Heatmap of sEV co-expression in AH from case 57 with total sample saturation. (B) Heatmap of sEV co-expression in AH from case 54 with titrated sample saturation.

1

2
